# Treatment Durations and Whitening Outcomes of Different Tooth Whitening Systems

**DOI:** 10.3390/medicina59061130

**Published:** 2023-06-12

**Authors:** Xiaoyi Zhao, Jie Pan, Hans Malmstrom, Yanfang Ren

**Affiliations:** 1 Department of General Dentistry & National Center of Stomatology & National Clinical Research Center for Oral Diseases & National Engineering Research Center of Oral Biomaterials and Digital Medical Devices, Peking University School and Hospital of Stomatology, Beijing 100081, China; xy724@163.com (X.Z.); panjie72@sina.com (J.P.); 2 Eastman Institute for Oral Health, University of Rochester Medical Center, Rochester, NY 14620, USA; hans_malmstrom@urmc.rochester.edu

**Keywords:** tooth whitening, in-office whitening, take-home whitening, treatment durations, dental enamel

## Abstract

*Background and Objectives*: Tooth whitening is a relatively conservative and effective option to treat discolored teeth. However, questions remain whether in-office or at-home tooth whitening products with short treatment durations are as effective and stable as products with longer treatment durations. *Materials and Methods*: Forty human third molars with intact enamel surfaces were divided into four groups of ten each, subjected to discoloration challenges with coffee for 60 h, and they were treated with four professional tooth whitening systems: two for take-home use—6% hydrogen peroxide for 30 min/d for a total of 7 h in 14 days (HP6), 10% carbamide peroxide for 10 h/d for 140 h in 14 days (CP10), as well as two for in-office use—35% HP for 10 min × 3 (HP35) for a total of 30 min and 40% HP for 20 min × 3 (HP40) for a total of 60 min. Teeth colors were assessed in the CIE L*a*b* color space with a spectrophotometer immediately and six months after whitening treatments. Surface roughness (Sa) for the treated and untreated enamel surfaces of the teeth in all groups were evaluated with a three-dimensional laser scanning microscope after six months. *Results*: No significant differences were found between HP6 and CP10 groups immediately after whitening (∆*E* 10.6 ± 1.6 vs. 11.4 ± 1.7, *p* > 0.05) and at six months after treatments (∆*E* 9.0 ± 1.9 vs. 9.2 ± 2.5, *p* > 0.05), or between HP35 and HP40 groups immediately after whitening (∆*E* 5.9 ± 1.2 vs. 5.3 ± 1.7, *p* > 0.05) and at six months after treatments (∆*E* 7.2 ± 1.6 vs. 7.7 ± 1.3, *p* > 0.05). The two at-home whitening systems achieved significantly better whitening outcomes than the two in-office products immediately after whitening (*p* < 0.05). However, at six months after treatments, the differences between at-home and in-office treatments had narrowed significantly (*p* > 0.05). There were no statistically significant differences with respect to the Sa values between the treated and untreated surfaces (*p* > 0.05). *Conclusions*: Tooth whitening products in the same product category have similar whitening efficacies, despite significant differences in treatment durations (7 vs. 140 h, and 30 min vs. 60 min, respectively). Take-home products achieved better whitening outcomes than in-office products, but they needed 14 to 280 times longer treatment durations.

## 1. Introduction

Vital tooth whitening is a popular option among dentists to treat discolored teeth due to its non-invasive, effective, safe, reliable, and low-cost properties [1]. Tooth whitening using various percentages of hydrogen peroxide (HP) or carbamide peroxide (CP) is a commonly used treatment that can be performed at home or in-office. The chromophore hypothesis has been used to explain the process of tooth whitening, which is primarily based on the interaction between HP and organic chromophores inside the tooth structure. As the whitening material diffuses across the surface of the tooth, it interacts with stain molecules on the surface and also affects the optical properties of the dental hard tissues within [2].

Whitening systems have been classified based on the type of whitening chemical employed, its concentration, method of administration, and frequency of applications. There are two main categories of professional tooth whitening products: at-home and in-office whitening systems. At-home whitening, using low concentrations of CP or HP in a custom tray, has been shown to be efficient in obtaining whiter teeth while causing no or very mild temporary tooth discomfort [3]. According to product directions, one or two applications per day are recommended, lasting 30 min to 2 h per session, or an overnight (8–10 h) application over a period of at least two weeks [4], which means at-home whitening procedures may vary greatly in treatment durations, lasting 7 h to 140 h in total. Some studies have shown better whitening outcomes with longer treatment times [5], while others have shown that treatment durations had no such effect [3]. During in-office whitening, higher concentrations of oxidative agents are used for shorter periods of time. The concentration of HP in in-office whitening agents may vary from 25% to 40% [6,7]. A whitening effect may be seen within 30 to 60 min after one treatment session [8]. The relationship between treatment durations and whitening outcomes remains unclear for in-office whitening, with some showing that whitening effects were dependent on treatment durations, but not on HP concentrations [9], while others showed that HP concentrations and application methods played important roles in whitening outcomes [10].

One of the major concerns after whitening is the long-term stability of the whitening outcomes. Color regression was frequently reported in short term studies in vitro [10,11]. Dehydration of the teeth may cause transient whitening during treatment, and there was significant reduction in color changes at one to six weeks after treatment [9]. Though the whitening system, whitening agent, and duration of treatment might affect the stability of the whitening outcomes [12], the presence of mineral content in the oral environment was another contributing factor to color regression after whitening treatment [13]. Different patterns of response after whitening have been described in studies in vivo, as compared to studies in vitro. Though some laboratory studies showed significant regression in whitening effects in the first week after treatment for both in-office and at-home whitening [14], clinical studies with longer follow-up durations found that whitening outcomes did not change over a six-month period [15]. Another study showed an improved whitening effect three months after in-office whitening in a Chinese population [16]. It is not known if the discrepancies in color regression between studies in vitro and those in vivo were due to the differences in follow-up time. No studies in vitro have assessed the color stability at six months after whitening treatment.

Questions remain whether in-office or at-home tooth whitening products with short treatment durations are as effective and stable as products with longer treatment durations. Therefore, the primary aim of the current study was to examine the whitening outcomes after treatments with different whitening systems that require significantly different treatment durations; and, the secondary aim was to assess the effects of different treatment systems on enamel morphology and enamel surface roughness. We hypothesized that the same category of whitening systems with different treatment durations produce the same immediate and long-term whitening effects.

## 2. Materials and Methods

A total of 40 freshly extracted permanent third molars were collected from general dentistry clinics of Peking University School and Hospital of Stomatology, in accordance with the guidelines of the Institutional Review Board of the authors’ institution (approval number: PKUSSIRB-202054043). The teeth were cleaned of soft tissues and stored in 0.1% thymol solution for no more than two months before use.

### 2.1. Sample Preparation

All the specimens were exposed for 60 h to a coffee solution prepared with 1.8 g coffee (Nescafe Rich Blend, Vevey, Switzerland; pH 5.38) dissolved in 150 mL of boiling water and cooled to room temperature. After discoloration challenges in the coffee solution, the buccal and lingual surfaces of the teeth were brushed each, for 20 strokes, under a pressure of 200 g with a manual toothbrush (American Dental Association standard toothbrush with soft bristles, provided by the American Dental Association) and a toothpaste containing hydrated silica abrasives (Colgate Cavity Protection Regular Flavor, Colgate-Palmolive) to ensure that all colorants adsorbed on the teeth surface were removed.

**Figure 1:**
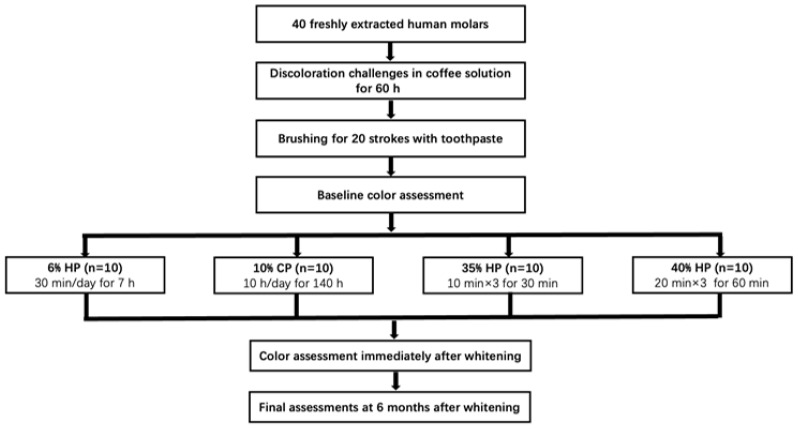
Flow diagram of the experimental design. Specimens were stored in artificial saliva between measurements. HP: hydrogen peroxide. CP: carbamide peroxide.

Specimens were mounted on a small silicone rubber base to allow the buccal surfaces to face upwards. The buccal surface underwent whitening treatment, while the lingual surface was untreated. A flow diagram of the experimental design is shown in Figure 1.

### 2.2. Whitening Treatments

The teeth (N = 40) were divided into four groups of 10 each after discoloration challenges with coffee and after brushing and treated with 4 professional whitening systems, following the manufacturer’s instructions (Table 1). The specimens were kept in artificial saliva at 37 °C and pH 7 after whitening treatment. The composition of the artificial saliva was described by Zhao et al. [17] and contained the following chemicals in one liter of distilled water: 0.33 g KH_2_PO_4_; 0.34 g Na_2_HPO_4_; 1.27 g KCl; 0.16 g NaSCN; 0.58 g NaCl; 0.17 g CaCl_2_; 0.16 g NH_4_Cl; 0.2 g urea; 0.03 g glucose; and 0.002 g ascorbic acid. Artificial saliva was freshly prepared and changed every day during the experiment.

Group 1 (HP6), Beyond Corewhite ^TM^ (Beyond International Inc., Stafford, TX, USA) at-home whitening system, contained 6% HP. Discolored teeth (n = 10) were treated for 30 min a day for 14 days, for a total of 7 h in treatment durations.

Group 2 (CP10), Opalescence ^TM^ PF 10% (Ultradent Products Inc., South Jordan, UT, USA) at-home whitening system, contained 10% CP. Discolored teeth (n = 10) were treated 10 h a day for 14 days, for a total of 140 h in treatment durations.

Group 3 (HP35), Beyond Max5 ^TM^ (Beyond International Inc.) in-office whitening system, contained 35% HP. Discolored teeth (n = 10) were treated for 10 min per session for 3 sessions under the BEYOND Whitening Accelerator lamp head at a 90° angle to the teeth, for a total of 30 min in treatment duration.

Group 4 (HP40), Opalescence Boost ^TM^ (Ultradent Products Inc.) in-office whitening system, contained 40% HP. Discolored teeth (n = 10) were treated for 20 min per session for 3 sessions, for a total of 60 min in treatment duration.

### 2.3. Color Assessments

The color of the buccal surface was assessed in the Commission Internationale de l’Éclairege L*a*b* (CIE Lab) color space using an Olympus CrystalEye^®^ dental spectrophotometer (Olympus, Tokyo, Japan) [18]. The CIE Lab system is a chromatic value color space that measures the value and chroma on 3 coordinates. Color was measured after discoloration challenges and brushing (baseline), immediately after completion whitening treatments, and after 6 months of storage in artificial saliva for each group.

### 2.4. 3D Scanning for Surface Morphology and Surface Roughness Measurements

After 6 months, both the buccal (treated) and lingual (untreated) surfaces of the teeth in all groups were evaluated with a 3D laser scanning microscope (VK- X200, Keyence, Osaka, Japan). 3D images of the surfaces were obtained at magnifications of × 400 (see Figure 3 for typical images). Three areas of 530 μm × 700 μm each were selected, and the arithmetic mean heights of each area (Sa) were measured using the VK-X3000 Imaging Analysis software. Mean values of Sa were calculated from the 3 areas to represent the surface roughness of the treated and untreated surfaces.

### 2.5. Statistical Analysis

The sample size was estimated based on a pilot study with 35% HP for 30 min that resulted in a ∆E of 6.7 (±1.2). Based on this result and a perceptible threshold of tooth color difference at ∆E 1.6 for human eyes [19], we will need a sample size of 9 for each group to test the hypothesis with 80% power at an alpha level of 0.05. We decided to have 10 samples for each group. The primary outcome measure was the overall color difference (Δ*E*) of the treated surfaces after whitening, as well as after 6 months. A paired t-test was used to compare variables measured at different time points. One-way ANOVA with post hoc FLSD tests was used to compare the whitening outcomes among the study groups. Surface roughness were compared between the treated and untreated surfaces (paired *t*-test) and among groups (ANOVA). The significance level for all statistical analyses was set at 0.05.

## 3. Results

### 3.1. Color Changes after Whitening Treatments

**Figure 2:**
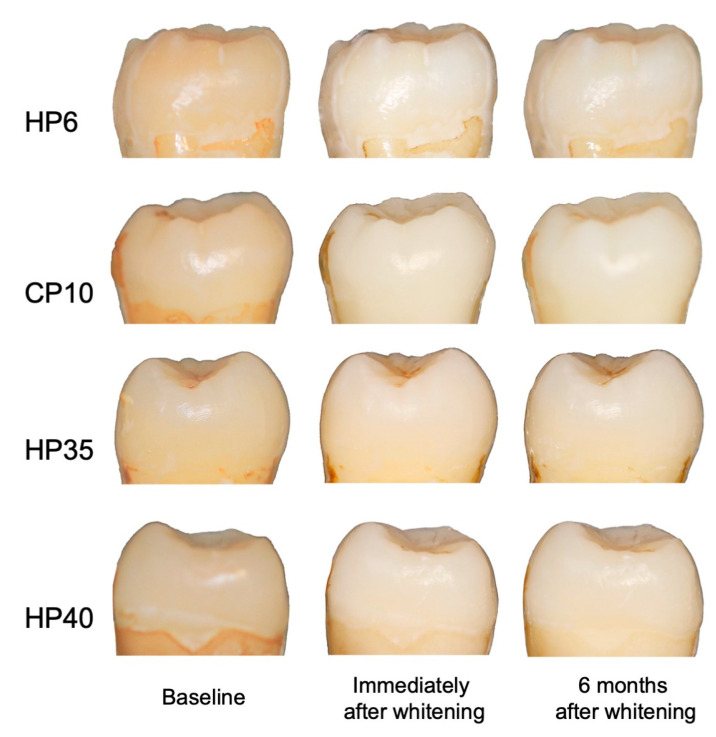
Representative tooth color in the four study groups at baseline, immediately after completion of whitening treatment, and six months after whitening treatment.

The color change and the mean L*, a*, and b* values for each group were shown in Figure 2 and Table 2. At baseline, there were no differences in the CIE Lab color space among the study groups (*p* > 0.05). All four whitening systems resulted in significant increase in lightness (higher L*) (*p* < 0.05), as well as reductions in redness and yellowness (lower a* and b*) (*p* < 0.05) immediately after the completion of the whitening treatments. After six months, L* values decreased in HP6 (t = 4.19, *p* < 0.05) and CP10 (t = 7.19, *p* < 0.05) groups, while they increased in HP35 (t = −3.93, *p* < 0.05) and HP40 (t = −5.15, *p* < 0.05) groups. No significant differences of L* value among the four study groups were found after six months (*p* > 0.05).

As shown in Table 3, color changes (Δ*E* values) after at-home whitening in HP6 group were 10.60 ± 1.62, compared to 11.38 ± 1.69 in CP10 group. There were statistically significant reductions in ΔE values after six months for both the HP6 and CP10 groups, at 9.04 ± 1.86 (t = 4.82, <0.05) and 9.19 ± 2.52 (t = 5.59, <0.05), respectively. No significant differences were found between HP6 and CP10 groups immediately after whitening treatments (t = −1.05, *p* > 0.05) and at six months after treatment (t = −0.15, *p* > 0.05).

The Δ*E* values for in-office whitening immediately after treatments were 5.92 ± 1.19 for HP35 and 5.28 ± 1.74 for HP40 groups, respectively. There were statistically significant increases in *ΔE* values after six months for both the HP35 and HP40 groups, at 7.20 ± 1.61 (t = −6.19, <0.05) and 7.68 ± 1.30 (t = −6.05, <0.05), respectively. No significant differences were found between HP35 and HP40 groups immediately after whitening treatments (t = 0.97, *p* > 0.05) and at six months after treatments (t = −0.74, *p* > 0.05).

As shown in Table 3, the two at-home whitening systems achieved significantly better whitening outcomes than the two in-office products immediately after whitening (F = 39.69, *p* < 0.05). However, at six months after treatments, the differences between at-home and in-office treatments had narrowed significantly (F = 2.74, *p* > 0.05).

### 3.2. Surface Roughness of Treated and Untreated Enamel Surfaces

**Figure 3:**
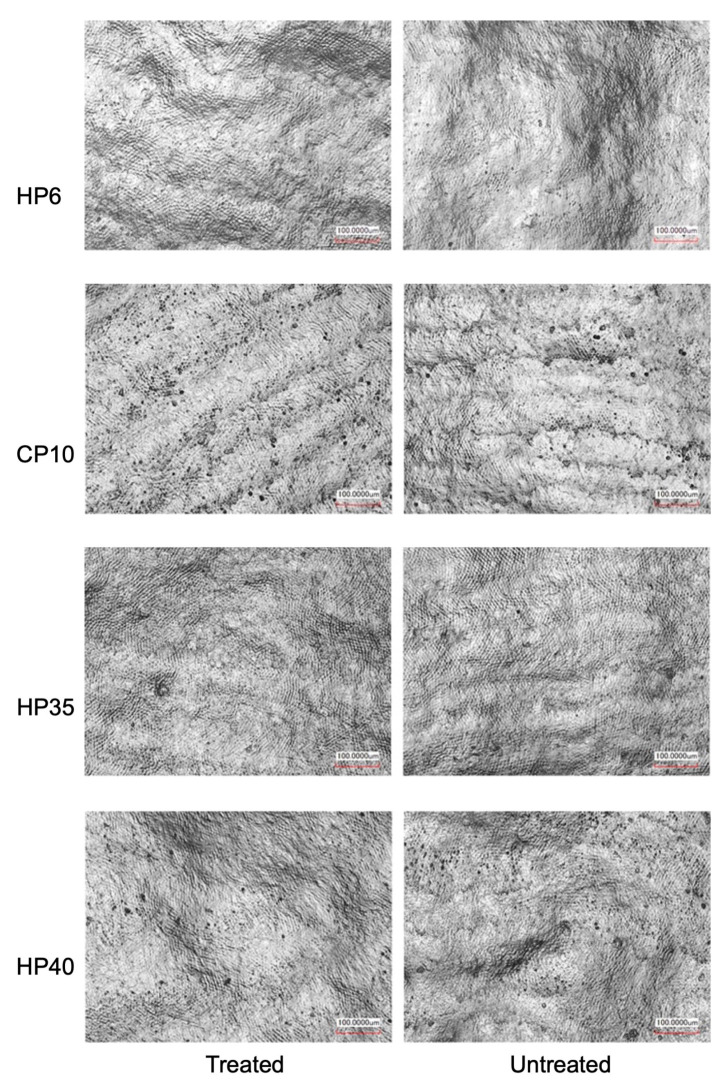
Morphology of treated and untreated enamel surfaces in the four study groups (×400, scale bar = 100 µm). No differences were found in enamel surface morphology between treated and untreated enamel surfaces in all four groups. HP: hydrogen peroxide. CP: carbamide peroxide.

Surface morphologies and surface roughness (Sa values) for the treated and untreated enamel surfaces at six months after whitening treatment are shown in Figure 3 and Table 4. Whitening treatments did not result in visible changes in surface morphology at 400 × magnification (Figure 3). No statistically significant differences were found among the four treatment groups with respect to the Sa values between the treated and untreated surfaces (*p* > 0.05) (Table 4).

## 4. Discussion

The findings of the present study indicate that both at-home whitening systems produced similar tooth whitening effects, but they required significantly different length in treatment durations. The HP6 group achieved whitening outcomes after 7 h of total treatment time comparable to those of the CP10 group after 140 h. Similarly, the two in-office whitening systems also achieved comparable tooth whitening outcomes, with HP35 requiring 30 min per session, half the time of HP40. These findings support the study’s first hypothesis that whitening systems within the same category produce similar whitening outcomes, albeit the required treatment durations were significantly longer for some products than others.

One explanation for the differences in treatment durations lies in the concentrations of the active whitening ingredient. CP needs to be broken down into HP and urea, with a 3:1 ratio, before it could play its tooth whitening role [20]. The concentration of HP influences the amount of reactive oxygen species available to oxidize the organic structure of dental tissues [2], which in turn affects the effectiveness and speed of the whitening process. For at-home whitening protocols, a concentration of 10% CP is an equivalent of 3.5% HP [21]. The low concentration of active ingredient in the CP formulation means that a much longer treatment duration, up to 10 h per day [5], is required to achieve the desired whitening outcomes, as compared to higher concentrations of HP products. At-home systems with 6–10% HP in concentration were usually used for 30 to 60 min per day, as compared to 2 to 10 h per day for the 10% CP products. A recent clinical study showed that 10% HP at 30 min per day achieved similar short- and long-term whitening effects compared to 10% CP at 8 h per day for 14 days, with similar occurrence of complications of mild dentin sensitivity and gingival irritation in both groups [3]. The findings of the present study indicate that HP at lower concentration (6%) may achieve comparable whitening outcomes. Lowering the concentration from 10% to 6% has significant practical significance, as tooth whitening products with greater than 6% HP in concentration are prohibited in countries in the European Union [22].

For the in-office whitening systems, we found that the product with 35% HP and 30 min of treatment duration achieved similar outcomes to that with 40% HP and 60 min treatment duration. This finding was consistent with a previous study that showed that 25% HP for 45 min achieved similar whitening outcomes with 40% HP for 60 min [10]. One possible explanation was the use of lights that purportedly accelerate the whitening process. Both the current study and the above study used light irradiations in conjunction with the relatively lower concentrations of HP. Though the utility of light for tooth whitening remains controversial [8,23], one potential benefit of using light in an in-office whitening system is that the heat from the light may activate the HP and increase its breakdown rate and the number of free radicals available to oxidize complex organic compounds in tooth hard tissues [24]. Several studies have shown that light irradiation can increase the reactivity of HP, resulting in a shorter treatment time while achieving the same whitening outcomes [14,15,25].

One significant concern following in-office teeth whitening is the stability of whitening outcomes over time. Though previous studies in vitro have shown significant reductions in whitening outcomes within one to six weeks after in-office treatments with HP products [9,10], clinical studies with longer follow-up times indicated that the whitening outcomes were rather stable [15,16]. We extended the observation time to six months, and we found that minor reduction in whitening effect occurred only for the at-home whitening system, while the whitening outcomes was improved slightly for the in-office whitening system after six months in artificial saliva. The mechanism for such divergence in whitening outcomes between the at-home and in-office treatments in vitro over time was not known. One possibility is that the crystal structures and mineral reuptakes of surface enamel are different after long durations of treatment with low concentration of HP, as compared to much shorter durations of high concentrations of HP [13,26]. Further study is needed to confirm these findings and to determine the mechanisms of this phenomenon.

The findings of the present study also indicate that the at-home whitening with low concentrations HP and long treatment durations may achieve superior whitening outcomes compared to a single treatment with the in-office systems at high HP concentrations. It has been shown that longer application of low concentration HP resulted in greater reduction in enamel crystal size, as compared to short use of high concentration HP [26]. Enamel crystal size is inversely proportional to tooth lightness, and smaller enamel crystals scatter light more from the surface, causing the substrate to appear lighter [27]. This finding also implies that that prolonged application time may have a greater impact on enamel structure and composition than the HP concentration. However, such impact may not be clinically significant, as no differences in enamel surface morphology or roughness were found among the study groups. Other studies also found no differences in surface enamel roughness after treatment with different concentrations of HP products for the same duration [28]. The remineralizing action of saliva may have contributed to this effect [29]. Brushing the teeth with a mineralizing paste may also change the surface properties of the enamel after whitening treatment [30].

One significant limitation of the current study design is its inability to assess dentin sensitivity that may occur following both take-home and in-office tooth whitening treatments. The incidence of dentin sensitivity may be associated with treatment durations or concentrations of HP in the whitening products. A randomized controlled clinical trial design is needed to assess the effects of treatment durations and HP concentrations on dentin sensitivity.

## 5. Conclusions

Within the limitations of the current study in vitro, we conclude that tooth whitening products within the same product category demonstrated similar whitening outcomes, despite significant variations in treatment duration (7 vs. 140 h for take-home whitening, as well as 30 min vs. 60 min for in-office whitening). At-home products were found to achieve significantly better whitening outcomes than in-office products, but they required 14 to 280 times longer treatment durations. The differences in whitening outcomes between at-home and in-office treatments became less pronounced after six months. No changes were observed in enamel surface morphology or surface roughness after whitening treatments. These findings support that both the at-home and in-office whitening systems are effective, and patients may decide which system to use based on their personal preferences of treatment durations.

## Figures and Tables

**Table 1:** Commercial name, composition, manufacturer directions, and experiment application protocol.

Groups	Whitening Systems	Compositions	Manufacturer Directions *	Treatment Duration
HP6	Beyond Corewhite (Beyond International Inc. Stafford, TX, USA)	An amount of 6% hydrogen peroxide, de-ionized water, propylene glycon, stabilizers, carbomer, trolamine, sodium hydroxide, flavor, and sodium saccharin	Load the whitening gel to facial surface inside a custom tray, and wear the tray for 30 min per day	30 min/d, 14 d (total 7 h)
CP10	Opalescence TM PF 10% (Ultradent Products Inc, South Jordan, UT, USA)	An amount of 10% carbamide peroxide, glycerin, water, xylitol, carbomer, PEG-300, sodium hydroxide, EDTA, potassium nitrate, and sodium fluoride	Load the whitening gel into the deepest and outmost portion of custom tray, and wear the tray 8–10 h overnight	10 h/d, 14 d (total 140 h)
HP35	Beyond Max 5 (Beyond International Inc. Stafford, TX, USA)	An amount of 35% hydrogen peroxide, potassium nitrate, hydroxyapatite, propylene glycon, sodium hydroxide, and carbomer	Apply a 2–3 mm layer of to the dry surface. Position the Whitening Accelerator lamp head at a 90° angle to the teeth to begin the first 10 min cycle. Repeat for a total of 3 cycles.	10 min × 3 (total 30 min)
HP40	Opalescence Boost (Ultradent Products Inc, South Jordan, UT, USA)	An amount of 40% hydrogen peroxide, potassium nitrate, sodium fluoride, potassium hydroxide, carbopol, glycerin, and distilled water	Apply a 0.5–1.0 mm thick layer of gel to the labial surface of the tooth and slightly onto the incisal surface. Allow gel to remain on the teeth 20 min. Repeat for a total of 3 cycles.	20 min × 3 (total 60 min)

**Table 2:** Mean L*, a*, and b* values (± SD) before (baseline), immediately after, and six months after whitening treatments.

		HP6	CP10	HP35	HP40
Baseline	L*	68.89 ± 2.49	70.24 ± 1.80	69.59 ± 1.65	69.98 ± 1.21
	a*	3.51 ± 0.95	3.30 ± 0.53	3.20 ± 0.68	3.32 ± 0.60
	b*	18.83 ± 1.98	21.07 ± 3.30	19.41 ± 2.02	20.70 ± 3.59
After whitening	L*	76.80 ± 2.57	77.50 ± 1.81	73.75 ± 1.35	73.66 ± 0.81
	a*	0.86 ± 0.72	0.16 ± 0.53	2.61 ± 0.84	2.65 ± 0.47
	b*	12.54 ± 1.70	13.01 ± 2.70	15.29 ± 2.45	17.28 ± 2.47
After six months	L*	75.17 ± 3.02	74.35 ± 2.62	74.66 ± 1.37	75.48 ± 1.12
	a*	1.13 ± 0.90	0.06 ± 0.46	1.52 ± 0.71	1.13 ± 0.54
	b*	12.98 ± 1.62	13.73 ± 2.57	14.79 ± 2.72	16.04 ± 2.59

**Table 3:** Whitening outcomes (∆*E* ± SD) immediately after treatments and at six months after whitening in the four study groups.

	HP6	CP10	HP35	HP40
After whitening	10.60 ± 1.62 ^aA^	11.38 ± 1.69 ^aA^	5.92 ± 1.19 ^bA^	5.28 ± 1.74 ^bA^
After 6 months	9.04 ± 1.86 ^aB^	9.19 ± 2.52 ^abB^	7.20 ± 1.61 ^bB^	7.68 ± 1.30 ^abB^

Different letters within columns and lines indicate statistically significant differences (*p* < 0.05). Lowercases represent row differences, while uppercases represent column differences. HP: hydrogen peroxide. CP: carbamide peroxide.

**Table 4:** Surface roughness in μm (Mean Sa± SD) of treated and untreated surfaces at six months after whitening treatment in the four study groups.

	HP6	CP10	HP35	HP40
Treated	7.76 ± 1.13 ^aA^	6.44 ± 2.88 ^aA^	6.93 ± 1.27 ^aA^	8.72 ± 0.99 ^aA^
Untreated	6.01 ± 1.67 ^aA^	7.55 ± 2.29 ^aA^	7.62 ± 1.49 ^aA^	7.12 ± 2.31 ^aA^

Different letters within columns and lines indicate statistically significant differences (*p* < 0.05). Lowercases represent row differences, while uppercases represent column differences. HP: hydrogen peroxide. CP: carbamide peroxide.

## Data Availability

The data presented in this study are all included in this article.
